# Clock-Controlled and Cold-Induced *CYCLING DOF FACTOR6* Alters Growth and Development in Arabidopsis

**DOI:** 10.3389/fpls.2022.919676

**Published:** 2022-07-26

**Authors:** Emily J. Blair, Greg S. Goralogia, Matthew J. Lincoln, Takato Imaizumi, Dawn H. Nagel

**Affiliations:** ^1^Department of Botany and Plant Sciences, University of California, Riverside, Riverside, CA, United States; ^2^Department of Biology, University of Washington, Seattle, WA, United States

**Keywords:** cold stress, circadian clock, *CDF6*, transcription factor, vasculature, abiotic stress

## Abstract

The circadian clock represents a critical regulatory network, which allows plants to anticipate environmental changes as inputs and promote plant survival by regulating various physiological outputs. Here, we examine the function of the clock-regulated transcription factor, CYCLING DOF FACTOR 6 (CDF6), during cold stress in *Arabidopsis thaliana*. We found that the clock gates *CDF6* transcript accumulation in the vasculature during cold stress. *CDF6* mis-expression results in an altered flowering phenotype during both ambient and cold stress. A genome-wide transcriptome analysis links CDF6 to genes associated with flowering and seed germination during cold and ambient temperatures, respectively. Analysis of key floral regulators indicates that CDF6 alters flowering during cold stress by repressing photoperiodic flowering components, *FLOWERING LOCUS T* (*FT*), *CONSTANS* (*CO*), and *BROTHER OF FT (BFT)*. Gene ontology enrichment further suggests that *CDF6* regulates circadian and developmental-associated genes. These results provide insights into how the clock-controlled CDF6 modulates plant development during moderate cold stress.

## Introduction

The circadian clock consists of an expansive regulatory network, which enables eukaryotic organisms to synchronize their metabolism, physiology, and development to daily and seasonal environmental changes (Greenham and McClung, 2015; Creux and Harmer, 2019). Through a coordinated and interconnected series of transcriptional–translational feedback regulations between multiple components, the clock modulates the expression of a large proportion of the transcriptome in plants. For example, the clock regulates 40–50% of genes involved in plant abiotic stress responses (Covington et al., 2008). Several recent transcriptome studies indicate that the time of day impacts the plant transcriptional response to abiotic stimulus (Wilkins et al., 2010; Blair et al., 2019; Grinevich et al., 2019; Bonnot et al., 2021; Markham and Greenham, 2021). The clock is also involved in regulating several critical developmental phenotypes. For example, mis-expression of some clock components (*CIRCADIAN CLOCK ASSOCIATED 1/CCA1, EARLY FLOWERING 3/ELF3, PSEUDO RESPONSE REGULATOR 9/PRR9, TIMING OF CAB EXPRESSION 1/TOC1, etc*.) results in altered hypocotyl growth and flowering (Nagel and Kay, 2012; Huang and Nusinow, 2016; Nakamichi, 2020).

In Arabidopsis, the clock coordinates aspects of photoperiodic flowering primarily through the regulation of GIGANTEA (GI) (Song et al., 2015). During long-day (16-h light: 8-h dark) conditions, GI forms a complex with FLAVIN-BINDING, KELCH REPEAT, F-BOX 1 (FKF1) to target the degradation of *CYCLING DOF FACTORs* (*CDFs*), a small subfamily of the DNA-binding with one finger (DOF) transcription factor (TF) family, which enables *CONSTANS* (*CO*) and *FLOWERING LOCUS T* (*FT*) to accumulate and promote flowering (Imaizumi et al., 2005; Sawa et al., 2007; Fornara et al., 2009; Nohales et al., 2019). However, during short-day conditions, the CDFs bind to the DOF-binding sites (AAAG) of *FT* and *CO* and act redundantly to suppress the accumulation of *FT* and *CO* mRNAs, resulting in delayed flowering (Imaizumi et al., 2005; Fornara et al., 2009). Recent work indicates that the vasculature-expressed *CDF6* negatively regulates *FT* transcript abundance, resulting in delayed flowering (Krahmer et al., 2019). CDF6 also interacts with GI, consistent with the known mechanism for the GI-FKF1 module degrading CDF family members during long-day conditions (Krahmer et al., 2019).

The *CDFs* are regulated by several clock components. For example, the day-expressed PRR9, PRR7, and PRR5 sequentially bind to the *CDF5* promoter region to repress its accumulation during the day (Martín et al., 2018). PRR7 and PRR5 associate with the promoter region of *CDF3* (Nakamichi et al., 2012). Furthermore, PRR9, PRR7, and PRR5 negatively regulate the expression of *CDF1*, while CCA1 positively regulates the *CDF1* expression *via* regulation of *GI* (Nakamichi et al., 2007). The expression of both *CDF5* and its natural antisense transcript, *CDF5 LONG NONCODING RNA (FLORE)*, is altered in *CCA1* over-expression lines (Henriques et al., 2017). In addition, the expression of the lesser characterized *CDF6* is also clock-regulated, and PRR9 and LATE ELONGATED HYPOCOTYL (LHY) directly bind to the *CDF6* promoter (Liu et al., 2016; Adams et al., 2018; Blair et al., 2019).

Photoperiodic flowering regulators are involved in various abiotic stress responses. GI is required for the drought escape, oxidative stress, and cold stress response (Riboni et al., 2013; Fornara et al., 2015). CDF3 confers tolerance to drought and freezing temperatures, while acting in both GI-dependent and GI-independent abiotic stress response pathways (Corrales et al., 2017; Renau-Morata et al., 2020). Furthermore, plants over-expressing the tomato orthologs of *CDF1* and *CDF3* show increased tolerance during drought and salinity stress (Corrales et al., 2014). *CDF1, 2, 3, 5*, and *6* are uniformly upregulated in response to cold stress (Kilian et al., 2007; Calixto et al., 2018; Blair et al., 2019). As such, the CDFs are considered part of the cold-regulated (*COR*) genes, due to their altered expression in a transcriptome analysis of the central cold regulators, *C-REPEAT BINDING FACTORS (CBFs)* triple loss-of-function mutants *(cbf123)* (Shi et al., 2017; Song et al., 2021). Interestingly, both *CDF5* and *CDF6* were induced over 2-fold times in response to low temperature in parallel with CBF1, CBF2, and CBF3 (Park et al., 2015). While *CDF3* confers cold stress tolerance and *CDF5* impacts hypocotyl elongation during short days (Corrales et al., 2017; Martín et al., 2020), the *CDFs* have not been shown to have differential functions in regulating plant growth and development during cold stress.

Here, we use a combination of meta-data, transcriptomic, genetic, and phenotypic approaches to better understand the interplay between the clock regulation and the function of the lesser characterized family member, CDF6, during cold stress. We find that CCA1 modulates *CDF6* transcript accumulation in response to moderate cold stress. Through analysis of tissue-specific (*SUC2*) and mutant genotypes, we find that *CDF6* influences photoperiodic flowering in both ambient and cold temperatures, and germination during ambient temperature. Specifically, vasculature-expressed *CDF6* represses *FT, CO*, and *BFT* to regulate flowering. Finally, we show that *CDF6* significantly alters the transcriptome during cold stress including the expression of genes involved in flowering, rhythmic, and metabolic processes.

## Materials and Methods

### Plant Materials and Growth Conditions

*Arabidopsis thaliana* seeds were sterilized for 3–4 h (~100 mL of 6% sodium hypochlorite and ~4 mL of concentrated hydrochloric acid), plated on 1X Murashige and Skoog (MS) medium supplemented with 1.5% sucrose (w/v), and stratified in the dark for three nights at 4°C. Seeds were grown at a constant temperature of 22°C with ~90 μmol photons·s^−1^·m^−2^, in diurnal (12-h light: 12-h dark; LD) cycles for 8 days. For circadian and time of day experiments, seedlings were transferred to constant light (LL) for 2 days before sampling. Columbia-0 (Col-0) was used as the wild-type (WT) control. Clock genotypes (*cca1-1/lhy-21, CCA1-OX (35S::CCA1), SUC2::CCA1 #18*, and *prr7-3/prr9-1*) and *CDF* mis-expressed line (*SUC2::HA-CDF6 #11*) were previously characterized (Farré et al., 2005; Pruneda-Paz et al., 2009; Endo et al., 2014; Nagel et al., 2014; Krahmer et al., 2019). The *cdf12356* quintuple line was generated using the CRISPR/Cas9-based gene editing constructs in pKIR1.1 plasmids (Tsutsui and Higashiyama, 2017) containing *CDF1* (5'- GTTTGGCTGGACAATTACAC-3') and *CDF6* (5'-GTCTCAAGTTAGAGATACTC-3') gRNAs using the *cdf235* triple T-DNA mutants as a genetic background (Fornara et al., 2009). To generate *cdf1* and *cdf6* mutations, the *cdf1235* quadruple mutant was generated first, and then the *cdf6* mutation was induced by gene editing in the *cdf1235* quadruple to generate the *cdf12356* quintuple mutant (Supplementary Figure 1). The *cdf6* single mutant is a SALK T-DNA insertion mutant line (*SALK_010734*), which was genotyped to confirm homozygosity. The reduced *CDF6* expression level was validated *via* quantitative real-time PCR (qRT-PCR). To generate *pCDF6::CDF6-GUS* plants, the genomic copy with ~1.2 kb of the promoter and the coding sequence was PCR purified and cloned into pENTR d-TOPO (Invitrogen). LR Clonase II was used to perform the Gateway reaction with pMDC162 to create *pCDF6::CDF6-GUS*. The sequences were confirmed *via* Sanger sequencing [Institute for Integrated Genome Biology (IIGB) Genomics Core, University of California, Riverside (UCR)], and the vectors were transformed into WT plants with *Agrobacterium*-mediated transformation. Two independent T3 lines (Lines A and B) were selected for GUS assays. For cold treatment, seedlings were exposed for 1 h at 10°C and sampled at 4-h intervals (ZT12–ZT36) along with control samples grown at ambient temperature (22°C) after 8 days of entrainment in LD and 2 days in continuous light (LL). For the continuous cold experiments, plants were grown in long-day conditions (16-h light: 8-h dark) for 8 days at 22°C. Then the seedlings were maintained at 22°C or transferred to long-day conditions at 10°C for 2 days before sampling every 4 h.

### Quantitative Real-Time PCR

Seedlings were prepared and grown as described above. The total mRNA was isolated with the GeneJET Plant RNA Purification Kit (Thermo Fisher Scientific). cDNA was synthesized using 1 μg of total RNA and was reverse-transcribed with the iScript cDNA synthesis kit (Bio-rad). qRT-PCR was performed with SYBR Green Master Mix (Thermo Fisher Scientific) with the CFX384 Touch Real-Time PCR Detection System (Bio-Rad). Three biological and three technical replicates were analyzed. The relative expression was calculated against the housekeeping gene, *ISPOENTENYL-DIPOHSPHATE DELTA ISOMERASE II* (*IPP2*), using the ΔΔCq method. See Supplementary Table 1 for gene-specific primer sequences and qRT-PCR conditions used in this study.

### GUS Assay

For beta-glucuronidase **(**GUS) assays, plants were grown in long-day conditions (16-h light: 8-h dark), similar to the flowering time experiments described in the following section, and GUS staining was performed as previously described with the following modifications (Yang et al., 2018). Four seedlings were harvested in 1 mL of cold 90% (v/v) acetone and then vacuumed for 10 min. Seedlings were fixed at room temperature for 60 min. Acetone was replaced with ~500 μl of wash buffer (10 mM EDTA, 50 mM phosphate buffer (pH 7), 0.1% (v/v) Triton X-100, 1 mM potassium ferrocyanide, and 1 mM potassium ferricyanide in 20% methanol) on ice. After vacuuming three times for 10 min, wash buffer was replaced with ~500 μl of staining buffer [10 mM EDTA, 50 mM phosphate buffer (pH 7), 0.1% (v/v) Triton X-100, 1 mM potassium ferrocyanide, 1 mM potassium ferricyanide, 20% methanol, and 2 mM X-Gluc (Carbosynth Ltd, United Kingdom)]. Seeds were vacuum-infiltrated for 40 min and then incubated for 20 min for *pCDF6::CDF6-GUS* line A and ~16 h for line B at 37°C. After incubation, seedlings were washed and stored in 70% (v/v) ethanol until imaging on a Leica M165 FC stereoscope (Leica Microsystems CMS GmbH, Wetzlar, Germany) using white light.

### Flowering Time and Seed Germination Assays

For flowering time assays, seeds were stratified for 2–3 nights after sterilization and grown for ~10 days on 1X MS plates at ~90 μmol photons·s^−1^·m-^2^. Seedlings were transferred to soil and grown in long-day (16-h light: 8-h dark) or short-day (8-h light: 16-h dark) conditions at 22°C or 10°C as indicated in figure legends. Flowering time was measured as the mean number of rosette leaves for at least three independent replicates with n≥6 plants per genotype. Germination was defined as the emergence of the radicle and measured for three biological replicates of 75–100 seeds from WT, *cdf6, cdf12356*, and *SUC2::CDF6*, using a modified method of Nelson et al. (2009). Briefly, seeds were liquid sterilized with 70% (v/v) ethanol and 0.05% (v/v) Triton X-100 followed by incubations with 70% (v/v) and 95% (v/v) ethanol. Seeds were sprinkled on filter papers placed on 0.8% (w/v) Bacto-agar plates. After plating, seeds were immediately transferred to constant light conditions (μmol photons·s^−1^·m-^2^) at 22°C or 10°C.

### RNA-Sequencing Setup and Analysis

Three biological replicates of WT, *cdf6*, and *SUC2::CDF6* were grown as described above and sampled with lights on (Zeitgeber Time 0, ZT0) after 1 h of temperature treatment at 10°C along with control seedlings grown at 22°C. About 1 μg of total RNA was isolated and treated with DNAse I (Millipore Sigma). mRNA purification and libraries were prepared as described previously (Blair et al., 2019). Final libraries were purified by Ampure XP beads. Library quality was confirmed *via* Qubit 2.0 Fluorescence Reader (ThermoFisher Scientific) and Bioanalyzer 2100 (Agilent Genomics). The analysis pipeline was also described previously (Blair et al., 2019). Briefly, the sequencing analysis was performed at the UCR IIGB Genomics Core facility on the NextSeq500 (Illumina), which generated single-end 75-bp sequences. Reads were mapped to the TAIR10 genome using Hisat2, and limma.voom was used to determine differential gene expression (Law et al., 2014; Kim et al., 2015; H Backman and Girke, 2016). Differentially expressed genes (DEGs) were defined as genes with −1 > Log_2_ fold change > 1 and false discovery rate < 0.05. Gene ontology (GO) terms were assigned with a previously published pipeline, and flowering-related genes were identified using a previously published dataset (Bonnot et al., 2019; Kinoshita and Richter, 2020).

### Meta-Data Analysis

The transcription factor binding site motif analysis was conducted by inputting 500 bp upstream of the DOF-TF family members, from the TAIR bulk data download, into Find Individual Motif Occurrences (FIMO) (Grant et al., 2011; Le Hir and Bellini, 2013). The timing of peak expression (phase) was determined with the Phaser tool from the DIURNAL database (Mockler et al., 2007). The list of *COR* genes was obtained from an analysis of the *cbf1, cbf2, and cbf3* transcriptomes (Shi et al., 2017). The authors used Cuffdiff default parameters to normalize, perform statistical analysis, and identify differentially expressed genes from FPKM values; please refer to the “Methods” section of Shi et al. (2017) for additional details. The upstream clock regulators of DOF TFs were compiled from published Chromatin Immunoprecipitation-sequencing (ChIP-seq) datasets (Huang et al., 2012; Liu et al., 2013, 2016; Nagel et al., 2015; Kamioka et al., 2016; Adams et al., 2018). TFs that may bind to *CDF6 in vitro* were identified using the DNA Affinity Purification (DAP)-sequencing genome browser by selecting TF families, searching for AT1G26790 (*CDF6*) in the viewer, and checking for peaks that might indicate TF occupancy (O'Malley et al., 2016).

### Identifiers of Genes Referenced in This Study

*AT5G62430/CDF1, AT5G39660/CDF2, AT3G47500/CDF3, AT2G34140/CDF4, AT1G69570/CDF5, AT1G26790/CDF6, AT1G69572/FLORE, AT2G31230/ERF15, AT1G60960/ATIRT3, AT2G46790/PRR9, AT5G02810/PRR7, AT1G01060/LHY, AT2G46830/CCA1, AT5G15840/CO, AT1G65480/FT, AT1G07887, AT1G53480/MRD1, AT1G53490/HEI10, AT1G76960, AT5G35935, AT5G35940, AT1G60960/ATIRT3, AT1G75945, AT2G06995, AT3G59930, AT5G33355, AT1G68050/FKF1, AT1G68840/ATRAV2, AT1G80340/GA3OX2, AT2G45660/SOC1, AT5G24470/PRR5, AT5G60910/AGL8*, and *AT5G62040/BFT*.

### Data Availability

The sequences reported in this paper have been deposited in the National Center for Biotechnology Information (NCBI) Gene Expression Omnibus (GEO) database and can be accessed through GEO Series accession GSE197581.

## Results

### CCA1 Gates *CDF6* Expression in Response to Cold Stress

**Figure 1:**
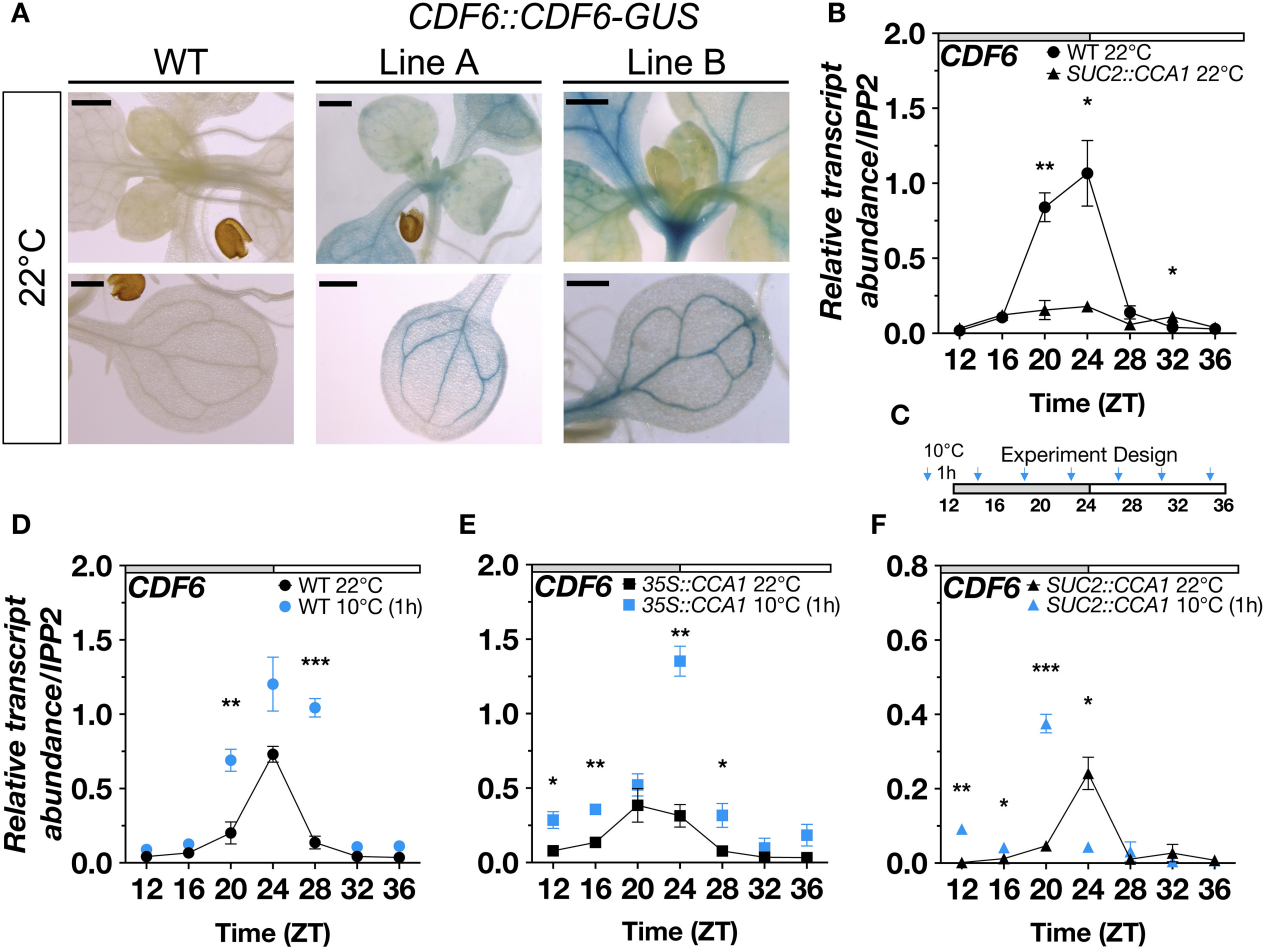
Vasculature-specific *CDF6* expression is gated by the clock in response to cold stress. **(A)** GUS activity in WT and two independent T3 *CDF6::CDF6-GUS* plants (Lines A and B) indicate the expression pattern of *CDF6* at ZT0 in 11-day-old seedlings grown in long-day (16-h light: 8-h dark) conditions. Bars correspond to 500 μm. **(B)** qRT-PCR of *CDF6* transcript abundance in WT (Col-0) and *SUC2::CCA1* in ambient temperature. qRT-PCR of *CDF6* transcript abundance in **(C)** continuous 22°C or 1 h of 10°C exposure in **(D)** WT, **(E)** *35S::CCA1*, and **(F)** *SUC2::CCA1*. Seedlings were grown in continuous light (LL) for 2 days after 8 days of entrainment in diurnal (12-h light:12-h dark; LD) cycles. Gray and white bars indicate the subjective night and day periods, respectively. Time (ZT) represents hours. mRNA levels are normalized to *IPP2* (mean values ± standard error (SE), *n* = 3; ***P ≤ 0.001, **P ≤ 0.05, *P ≤ 0.01; unpaired student's *t*-test).

We previously showed that the expression of *CDF6* is altered in the *cca1lhy* clock mutants under diurnal (12-h light: 12-h dark; LD) conditions (Blair et al., 2019). In this study, we found that in constant light (LL) and temperature, *CDF6* expression is also altered in the *CCA1* over-expressor (*35S::CCA1*), *cca1lhy*, and *prr7prr9* mutants, which is evident by dampened transcript accumulation and changes in peak expression (Supplementary Figures 2A–C). Similar to the other CDFs, we observed that *CDF6* is localized to the vasculature (Figure 1A). We therefore assessed the expression of *CDF6* in previously published lines where *CCA1* is driven by the phloem companion cell (PCC)-specific promoter of *SUCROSE-PROTON SYMPORTER* (*SUC2::CCA1*) (Endo et al., 2014). Interestingly, when *CCA1* is expressed in the PCC, *CDF6* transcript accumulation is significantly altered relative to WT (Col-0) (Figure 1B) and to a higher extent compared to changes in *35S::CCA1*, specifically between ZT20 and ZT24 (Supplementary Figure 2A), the time points where the transcript levels of both *CDF6* and *CCA1* are at the highest in the WT (Figure 1B; Supplementary Figure 3A). These results indicate that CCA1 regulates *CDF6* transcript accumulation in the PCC of the vasculature.

A subset of DOF-TFs (*CDF1,2,3,5,6, ADOF1*) is *COR* genes based on transcriptomic analysis of *cbf* mutants (Shi et al., 2017). In response to moderate cold stress (10°C), *CDFs* are mostly upregulated during the morning (Blair et al., 2019**)**. *CDF6* is significantly upregulated at ZT1 but not at ZT6, while *CDF1* and *CDF3* are significantly upregulated at both times of day (Blair et al., 2019**)**. To determine whether CCA1 is responsible for *CDF6* mRNA accumulation during cold stress, we exposed WT, *35S::CCA1, SUC2::CCA1*, and *cca1lhy* seedlings to 10°C for 1 h before sampling every 4 h (ZT12–ZT36) and quantifying *CDF6* transcript abundance. Seedlings were entrained for 8 days in LD before transfer to continuous light (LL). Of note, both *CCA1* and its partially redundant partner, *LHY*, displayed increased transcript abundance in response to cold depending on the time of the day (Supplementary Figures 3A–D). In WT, we found that *CDF6* transcript abundance, similar to *CCA1*, was significantly elevated in response to cold at the time points surrounding dawn (ZT20 and ZT28) (Figures 1C,D; Supplementary Figure 3A). However, *CDF6* displayed higher transcript abundance in response to cold at dawn (ZT24), late morning (ZT28), and dusk (ZT12 and ZT16) in the *35S::CCA1* line (Figure 1E). While in the *SUC2::CCA1* line, we observed that *CDF6* exhibits higher transcript accumulation across the night period (ZT12–ZT20) but lower at dawn (ZT24) in response to cold (Figure 1F). Furthermore, *CDF6* induction in response to cold at ZT24 in WT (though not significant) and *35S::CCA1* was significantly reduced in *SUC2::CCA1* but restored in the *cca1lhy* mutants (Figures 1D–F; Supplementary Figure 2D). However, the induction of *CDF6* transcript abundance at ZT28 in WT, *35S::CCA1*, and *cca1lhy* was abolished in *SUC2::CCA1* (Figures 1D–F; Supplementary Figure 2D). A comparison between WT, *35::CCA1*, and *SUC2::CCA1* further supports the temporal mis-regulation of *CDF6* in response to cold (Supplementary Figure 2E). Of note, similar to what was observed at 22°C, *CDF6::CDF6-GUS* lines also showed vasculature protein accumulation at ZT0 after 1 h of exposure to 10°C (Supplementary Figure 3E). Together, these data suggest that the clock *via* CCA1 gates *CDF6* expression which in turn diminishes the normal cold induction of *CDF6* during the day and may promote cold induction during the subjective night.

### CDF6 Regulates Flowering and Seed Germination

**Figure 2:**
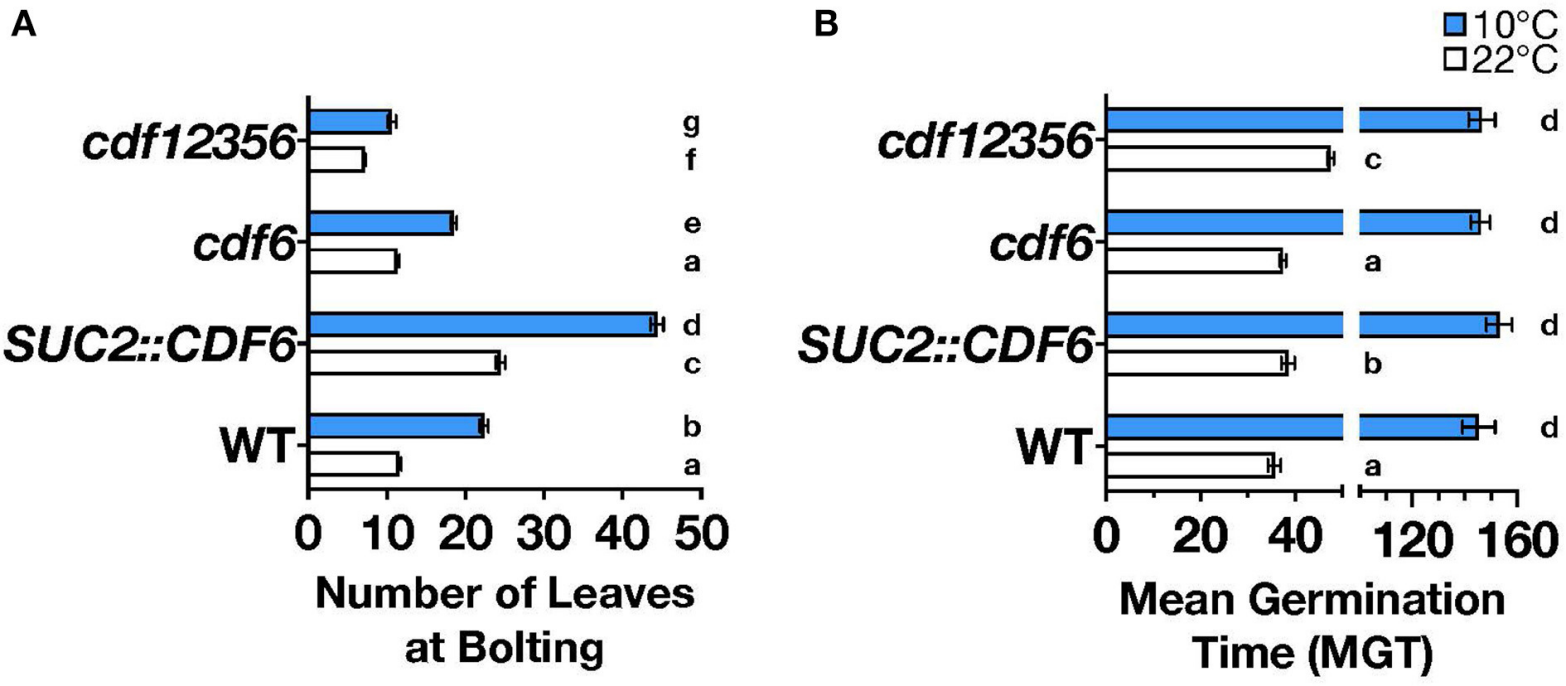
*CDF6* mis-expression alters flowering and seed germination. **(A)** The number of leaves at bolting for WT, *SUC2::CDF6, cdf6*, and *cdf12356* plants grown at constant 22°C (white bars) or 10°C (blue bars) in long-day conditions (mean values ± SE, *n* = 3+, two-way ANOVA with Tukey's multiple comparison test). **(B)** Mean germination time (MGT) for WT, *SUC2::CDF6, cdf6*, and *cdf12356* plants grown in constant light (LL) at 10°C or 22°C with lowercase a-c letters indicating statistical significance at 22°C and d at 10°C (mean values ± SE, *n* = 4, one-way ANOVA with Dunnett's multiple comparison test).

To date, higher orders of multiple mutants of *cdf1, cdf2, cdf3*, and *cdf5* are known to cause early flowering, and a more recent study demonstrated that *SUC2::CDF6* results in delayed flowering (Fornara et al., 2009; Krahmer et al., 2019). However, how cold stress impacts CDFs regulation of flowering time is still an open question. To examine further the role of *CDF6* in photoperiodic flowering during cold stress, we measured the flowering phenotype of *SUC2::CDF6* and a SALK T-DNA insertion line for *cdf6* (Supplementary Figures 4A,B), under continuous ambient (22°C) or cold (10°C) temperatures. As the CDFs share high-sequence homology and likely redundant function, we also measured flowering in the *cdf* quintuple mutant (*cdf12356*, Supplementary Figure 1). In long-day (16-h light: 8-h dark) conditions, the *SUC2::CDF6* plants displayed late flowering relative to WT under both ambient and cold temperatures (Figure 2A). Interestingly, the *cdf12356* mutant exhibits significant early flowering under both ambient and cold temperatures; however, this observed early flowering phenotype is notably reduced during cold (Figure 2A). While the *cdf6* single mutant did not show a significant flowering phenotype relative to WT at ambient temperature, under cold temperature, *cdf6* plants display a modest but significant early flowering phenotypes (Figure 2A). Furthermore, we observe a statistically significant interaction between temperature and genotype (Supplementary Figure 4C). These observations suggest that CDF6 plays a redundant role in the regulation of photoperiodic flowering during ambient temperature and a predominant role during cold temperatures. In short-day conditions (8-h light: 16-h dark), only *cdf12356* shows a difference relative to WT with early flowering in both cold and ambient temperatures (Supplementary Figure 4D).

*CDF4* and other DOF-TFs, such as *DOF AFFECTING GERMINATION1* (*DAG1*), *DAG2*, and *DOF6*, have been shown to play a role in germination (Ruta et al., 2020). To determine whether *CDF6* functions similarly to CDF4 and these other DOFs, we measured germination over time in constant light and temperature (22°C or 10°C) and in the *SUC2::CDF6, cdf6*, and *cdf12356* lines. *SUC2::CDF6* and *cdf12356* seeds have delayed germination relative to WT during ambient temperature but not under cold, whereas there is no significant difference in *cdf6* germination at either temperature (Figure 2B). Next, we measured seed dormancy after cold (4°C) and dark treatment of seeds for three nights and found germination synchrony across all genotypes tested (Supplementary Figures 4E,F). Thus, we conclude that during ambient temperature, *CDF6* may contribute to the regulation of seed germination rather than dormancy.

### Vasculature-Expressed *CDF6* Regulates the Transcriptome During Cold Stress

**Figure 3:**
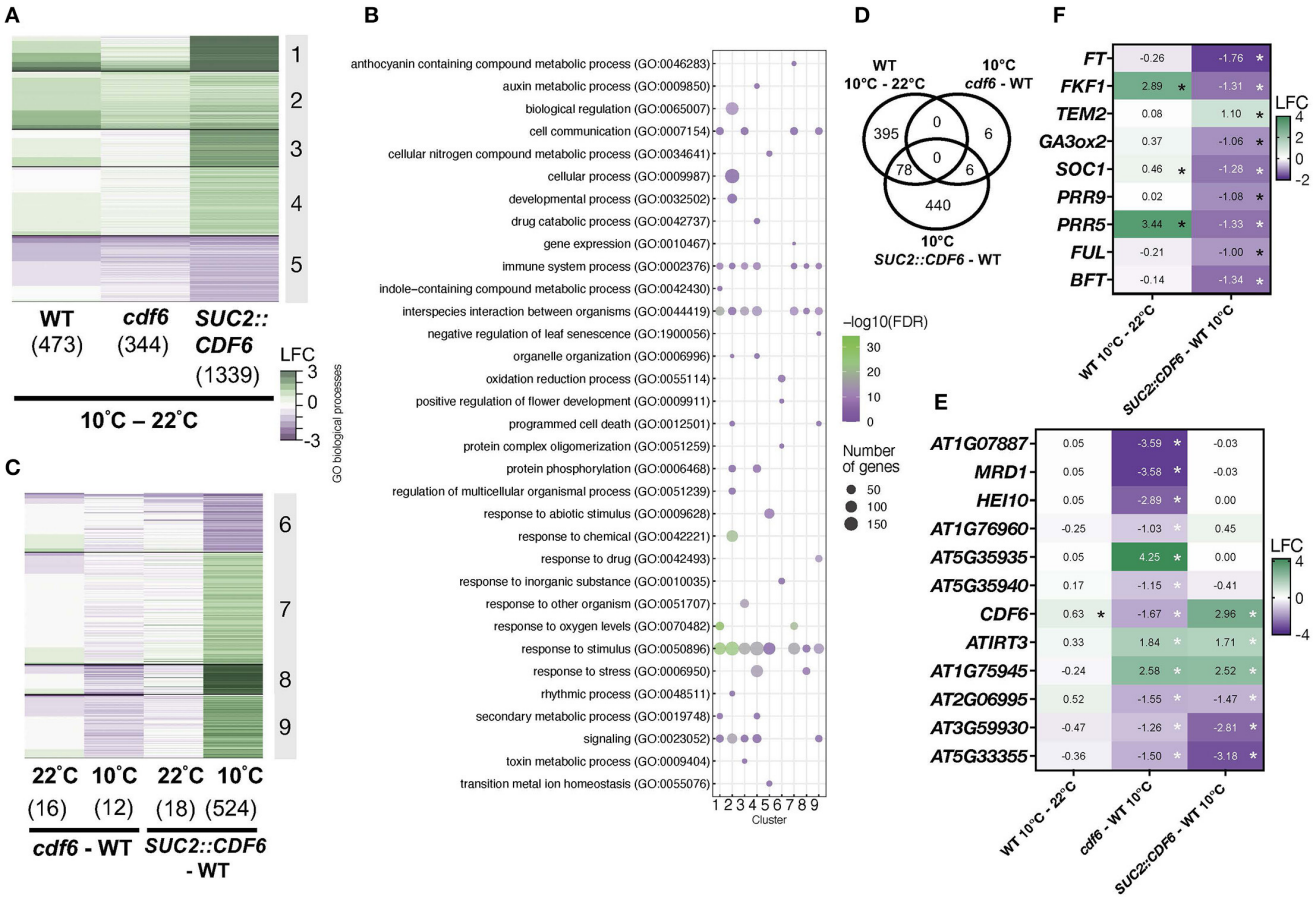
Vasculature-expressed *CDF6* regulates cold-responsive and flowering-associated genes in the morning. **(A)** Temperature and **(C)** genotype-specific DEGs [defined as −1 > Log_2_ fold change (LFC) > 1 and false discovery rate (FDR) < 0.05] identified after RNA-sequencing of three replicates of 11-day-old WT, *SUC2::CDF6*, and *cdf6* seedlings. Seedlings were grown in constant light for 2 days at 22°C after entrainment for 8 days in diurnal (LD) conditions before sampling at ZT0 after 1 h at 10°C or continuously at 22°C. **(B)** Summary of enriched GO biological process terms with indicated false discovery rate (FDR-corrected *p*-value < 0.05). GO terms selected based on a previously published protocol using a FDR cut-off < 0.05 (Bonnot et al., 2019). **(D)** DEGs specific to the cold response vs. *CDF6* mis-regulation during cold stress. Expression profiles of **(E)** 12 DEGs mis-expressed in *cdf6* and **(F)** flowering-associated genes identified in *SUC2::CDF6* during cold stress; *FDR < 0.05.

To determine a broader role for CDF6 in both development and cold responses, we examined transcriptome changes in WT and *CDF6* mis-expression lines (*SUC2::CDF6* and *cdf6*). For this, seedlings were entrained for 8 days in LD and then transferred to constant light (LL) for 2 days. On day 11, seedlings were subjected to 10°C for 1 h and sampled at subjective dawn (ZT0), the time of day *CDF6* expression is significantly altered in the *cdf6* mutant and *SUC2::CDF6* lines (Supplementary Figures 4B, 5F,G). Using a cut-off of −1 > Log_2_ fold change > 1 and false discovery rate (FDR) < 0.05, we identified 473 differentially expressed genes (DEGs) in the WT (10°C vs. 22°C), and we consider these as the generally cold-responsive DEGs in this study (Figure 3A, Supplementary Dataset 1). Of these generally cold-responsive genes, ~31% were previously identified as *COR* genes in the *cbf1, cbf2*, and *cbf3* mutants (Supplementary Dataset 2; Shi et al., 2017). Using the Phaser database which provides insight into clock regulation of transcript levels, we identified that ~50% of these DEGs exhibit rhythmic expression with significant enrichment from the afternoon to early evening period; this is consistent with the peak expression for cold-responsive genes and the proportion of the transcriptome that cycles during cold stress (Covington et al., 2008; Grundy et al., 2015; Supplementary Figure 5A; Supplementary Dataset 3).

Next, we identified 344 genes that are differentially expressed in *cdf6* at 10°C compared to 22°C; and ~71% of these are also found in the WT (10°C vs. 22°C) dataset (Figure 3A; Supplementary Figure 5B). However, the effect of *SUC2::CDF6* at 10°C compared to 22°C is most striking with 1339 DEGs being detected, and of these, only ~27% overlapped with the generally cold-responsive genes (Figure 3A; Supplementary Figure 5B). We speculate that the other ~73% of DEGs may be specifically regulated by *CDF6* in the vasculature in response to cold stress rather than across cell types in response to cold. A gene ontology (GO) analysis reveals enrichment for terms associated with the clock (Cluster 2), responses to abiotic and biotic factors (Clusters 1, 3, 4, and 5), and metabolic processes (Clusters 4 and 5) (Figure 3B). Cluster 2 also contains a number of flowering- and clock-related genes (Figure 3B).

To further dissect the impact of *CDF6* mis-expression on the transcriptome during cold stress, we compared each genotype relative to WT at each temperature. First, we observed that there are very few DEGs in *cdf6* compared to the WT (16 DEGs at 22°C and 12 DEGs at 10°C (Figure 3C). However, in vasculature-expressed *CDF6* (*SUC2:CDF6*), we observe a greater impact on the transcriptome during cold stress (524 DEGs) compared to ambient (18 DEGs) temperature (Figure 3C). Cluster 6 genes generally show downregulation in *SUC2::CDF6* compared to WT at 10°C with minimal expression change in the other comparisons. These genes were enriched for the biological GO term “positive regulation of flowering,” and many genes involved with flowering are found in this cluster, including the florigen molecule *FT* (Figure 3B). The other clusters show enrichment for terms associated with responses to stimulus/stress, immune system processes, and development (Figure 3B).

We also identified genes that may contribute to the delayed germination phenotype that we previously observed in *SUC2::CDF6* lines under ambient temperatures (Figure 2B; Supplementary Figure 5C). For example, IRON REGULATED TRANSPORTER 3 (ATIRT3) transports zinc, which is essential for the development of reproductive organs, and is needed for proper seed development (Lee et al., 2021). *IRT3* is upregulated in *SUC2::CDF6* at both temperatures, although this upregulation is reduced at 10°C when compared to at 22°C, suggesting that *SUC2::CDF6* seeds may have increased zinc transport to support germination during ambient temperature (Supplementary Figure 5C). In addition, *ETHYLENE RESPONSE FACTOR1*5 (*ERF15*), a positive regulator of ABA, is upregulated in *SUC2::CDF6* under both ambient and cold temperatures (Supplementary Figure 5C). Interestingly, *ERF15* over-expression results in delayed germination, similar to the phenotype we observed in *SUC2::CDF6* line (Lee et al., 2015). This suggests that vasculature-expressed *CDF6* may upregulate *ERF15* to delay germination and impact ABA responsiveness.

Next, we considered that a large number of DEGs could be generally cold-responsive rather than differentially expressed as a result of the absence of CDF6. Thus, we compared the 524 DEGs (*SUC2::CDF6*) and 12 DEGs (*cdf6)* to our generally cold-responsive DEGs and identified 6 DEGs specific to *cdf6* and 440 DEGs specific to *SUC2::CDF6* with an additional 6 shared by both *SUC2::CDF6* and *cdf6* genotypes (Figure 3D). The 12 *cdf6* DEGs perform various functions, including defense (*AT3G59930, AT5G33355*), class 1 crossover (*HEI10*), methionine biosynthesis (*MRD1*), and metal transport (*IRT3*), in addition to a handful of genes with unknown functions (Goto and Naito, 2002; Silverstein et al., 2005; Chelysheva et al., 2012; Lee et al., 2021) (Figure 3E). To further assess the extent of mis-expression due to cold stress rather than the effect of *CDF6*, we also compared the 473, 344, and 1,339 DEGs (shown in Figure 3A) at 10°C vs. 22°C in the WT, *cdf6*, and *SUC2::CDF6* genotypes, respectively. This analysis identified 61 DEGs that can be considered unique to *cdf6* (Supplementary Figure 5D), which are enriched for the following three biological GO terms: cellular response to heat, response to heat, and response to temperature stimulus (Supplementary Dataset 3). While these DEGs are not generally cold-responsive in comparison to our WT dataset, they may be generally temperature responsive. The other *CDFs* are not differentially expressed in *cdf6* or *SUC2::CDF6* at either temperature, with the exception of *CDF3* downregulation in *SUC2::CDF6* at 10°C (Supplementary Figure 5E). The data also confirm a significant depletion of *CDF6* mRNA in *cdf6* and elevation in *SUC2::CDF6*, both the response to cold and at ambient temperature, confirming this genotype elevates mRNA abundance in PCCs (Figure 3E; Supplementary Figures 5F,G). Future functional work with additional *cdf6* alleles and complementation lines is needed to define the regulatory mechanism of CDF6.

In the 440 *SUC2::CDF6-*specific DEGs at 10°C, we identified nine genes associated with flowering (*FT, FKF1, TEM2, GA3ox2, SOC1, PRR9, PRR5, FUL*, and *BFT*), all of which are downregulated with the exception of *TEM2* (Figure 3F). This complements previous reports of reduced expression in *SUC2::CDF6* in the evening under ambient temperature conditions (Krahmer et al., 2019). Interestingly, a few clock- or circadian-regulated genes, specifically *PRR9, PRR5*, and *FKF1*, are differentially expressed in *SUC2::CDF6* compared to WT at 10°C, indicating that there may be feedback regulation between *CDF6* and these components (Figure 3F). Many other flowering-related components identified are either TFs or involved in hormone signaling (Kinoshita and Richter, 2020; Supplementary Dataset 4).

### *CDF6* Alters the Expression of Key Flowering Genes During Cold Under Long-Day Conditions

**Figure 4:**
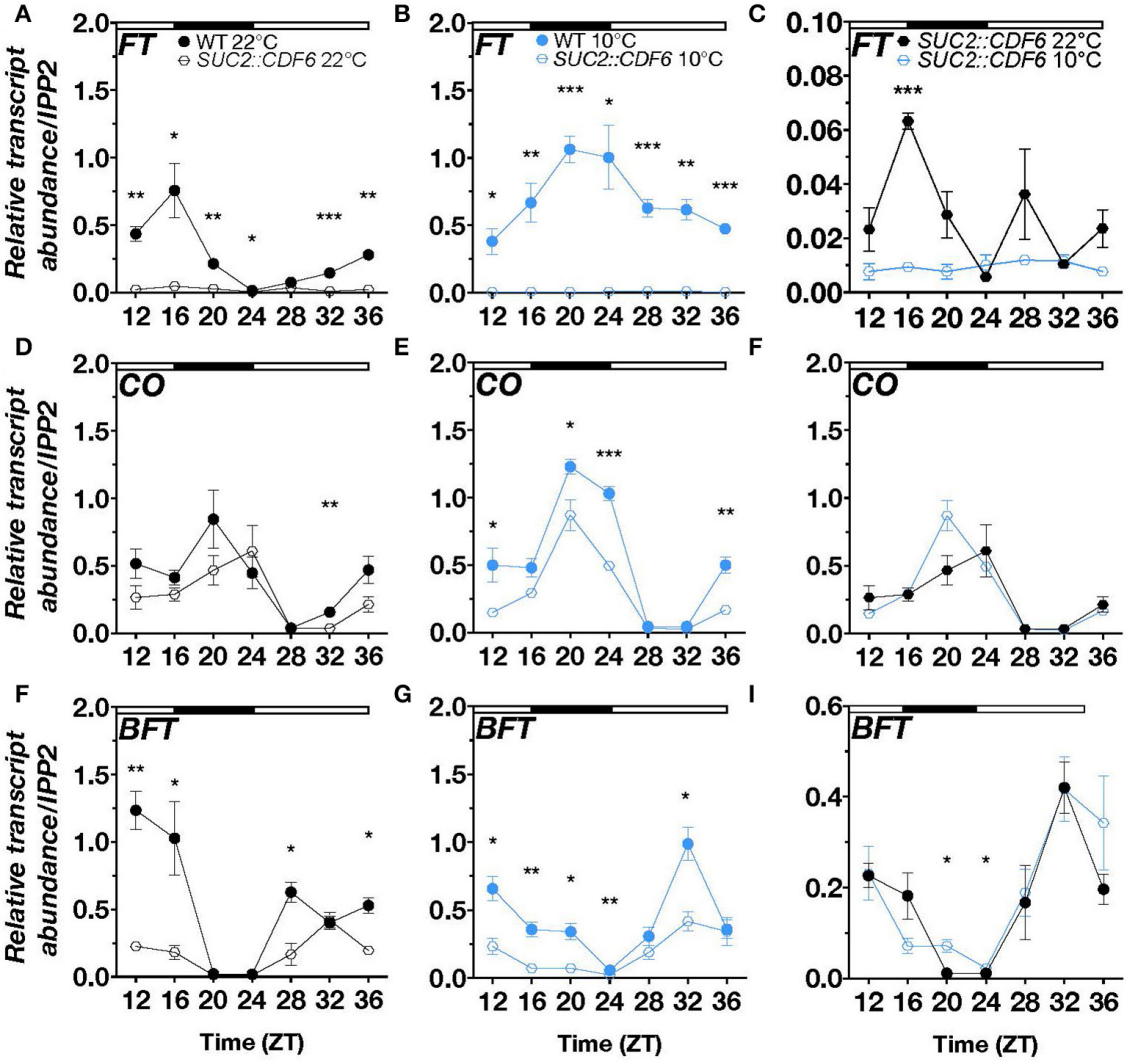
CDF6 alters *FT, CO*, and *BFT* expression during cold stress. qRT-PCR of **(A–C)** *FT*, **(D–F)** *CO*, and **(G–I)** *BFT* relative transcript abundance in WT and *SUC2::CDF6* seedlings. Plants were grown in long-day conditions at 22°C for 8 days followed by 2 days continuously at 22°C or 10°C. Seedlings were sampled every 4 h starting at 12 h after dawn. **(C)** *FT* **(F)** *CO*, and **(I)** *BFT* transcript abundance in *SUC2::CDF6* at 10°C and 22°C from the data shown in A and B, D and E, and G and H, respectively. White and black bars indicate the day and night periods, respectively. mRNA levels are normalized to *IPP2* (mean values ± SE, *n* = 3; ***P ≤ 0.001, **P ≤ 0.01, *P ≤ 0.05; unpaired student's *t*-test).

To better understand how *CDF6* regulates photoperiodic flowering during cold, we measured the abundance of the photoperiodic flowering component mRNAs, *FT, CO*, and *BFT, via* qRT-PCR over a 24-h period in continuous 10°C or 22°C long-day conditions. In WT and under constant cold, *FT* transcript accumulation is significantly increased from ZT20 to ZT36 and displays a shift in peak expression to ZT20 compared to its peak expression at ZT16 during ambient temperature (Supplementary Figure 6A). In *SUC2::CDF6* lines, we observed a significant reduction in *FT* transcript abundance relative to WT in the evening (ZT16 and ZT20), at dawn (ZT24), and late afternoon (ZT32, ZT12, and ZT36) (Figure 4A). At 10°C, *FT* accumulation is reduced relative to WT throughout the 24-h period (Figure 4B). Interestingly, in *SUC2::CDF6, FT* repression was significantly enhanced during the cold at ZT16, further supporting a distinct role for CDF6 in regulating photoperiodic flowering during cold stress (Figures 2A, 4C).

Under ambient temperature, *CO* shows a shift in peak expression from ZT20 to ZT24 in *SUC2::CDF6* relative to WT and is significantly downregulated in *SUC2::CDF6* at ZT32 (Figure 4D). *CO* has peak expression at ZT20 under continuous cold conditions, and is significantly repressed in the early morning (ZT20), at dawn (ZT24), and late evening (ZT12 and ZT36) in *SUC2::CDF6* compared to WT (Figure 4E). In addition, *CO* is significantly upregulated in response to cold in WT at ZT24, so it is likely that *pSUC2*-driven expression of *CDF6* prevents *CO* accumulation during the cold at this time point (Figure 4E, Supplementary Figure 6B). Although the repressive effect of *SUC2::CDF6* is not significantly different at 10°C compared to 22°C, our data together indicate that *CDF6* expression is required for appropriate accumulation of *CO* in WT at specific time points under both temperatures (Figure 4F). These findings are consistent with the transcriptome analysis, which shows that *FT* and *CO* are strongly downregulated during cold stress when *CDF6* is regulated by *pSUC2* (Figures 3D, 4). *FT* and *CO* are not significantly different in *cdf6* compared to the WT at either temperature tested, likely due to redundancy by other *CDFs* in photoperiodic flowering regulation (Supplementary Figures 6C,D).

Besides *FT, CO*, and the two clock components *PRR9* and *PRR5, BFT* is specifically localized to the PCC under control conditions (Supplementary Figure 7). In addition, transcriptome analysis revealed *BFT* to be downregulated in *SUC2::CDF6* seedlings during cold stress (Figure 3F). Therefore, we also assessed *BFT* transcript accumulation during continuous cold under long-day conditions in *SUC2::CDF6*. At ambient temperature, *CDF6* downregulates *BFT* during the day (ZT12, ZT16, ZT28, and ZT36) (Figure 4G). However, during continuous cold, *BFT* transcript abundance is significantly reduced in *SUC2::CDF6* during both the day and night periods (ZT12-ZT24 and ZT32) (Figure 4H). Together, these data indicate that *CDF6* represses *BFT* independent of temperature, although *BFT* mRNA accumulation is significantly increased in *SUC2::CDF6* in the early morning (ZT20 and ZT24) during continuous cold compared to ambient temperature (Figure 4I).

## Discussion

Previous work shows that the clock regulates a range of developmental programs, including germination, flowering, and senescence, to promote optimal survival and reproduction (Lu et al., 2012; Adams et al., 2018; Kim et al., 2018; Zha et al., 2019; Kyung et al., 2021). The clock is also known to regulate responses to abiotic stress. A classic example involves the clock gating of *CBF1-3* accumulation during cold stress, which promotes the activation of *COR* genes to confer increased cold tolerance (Fowler and Thomashow, 2002; Gilmour et al., 2004; Fowler et al., 2005). Additional evidence suggests that the expression of *CBFs* is regulated by PRR5, PRR7, and PRR9 (Nakamichi et al., 2009).

**Figure 5:**
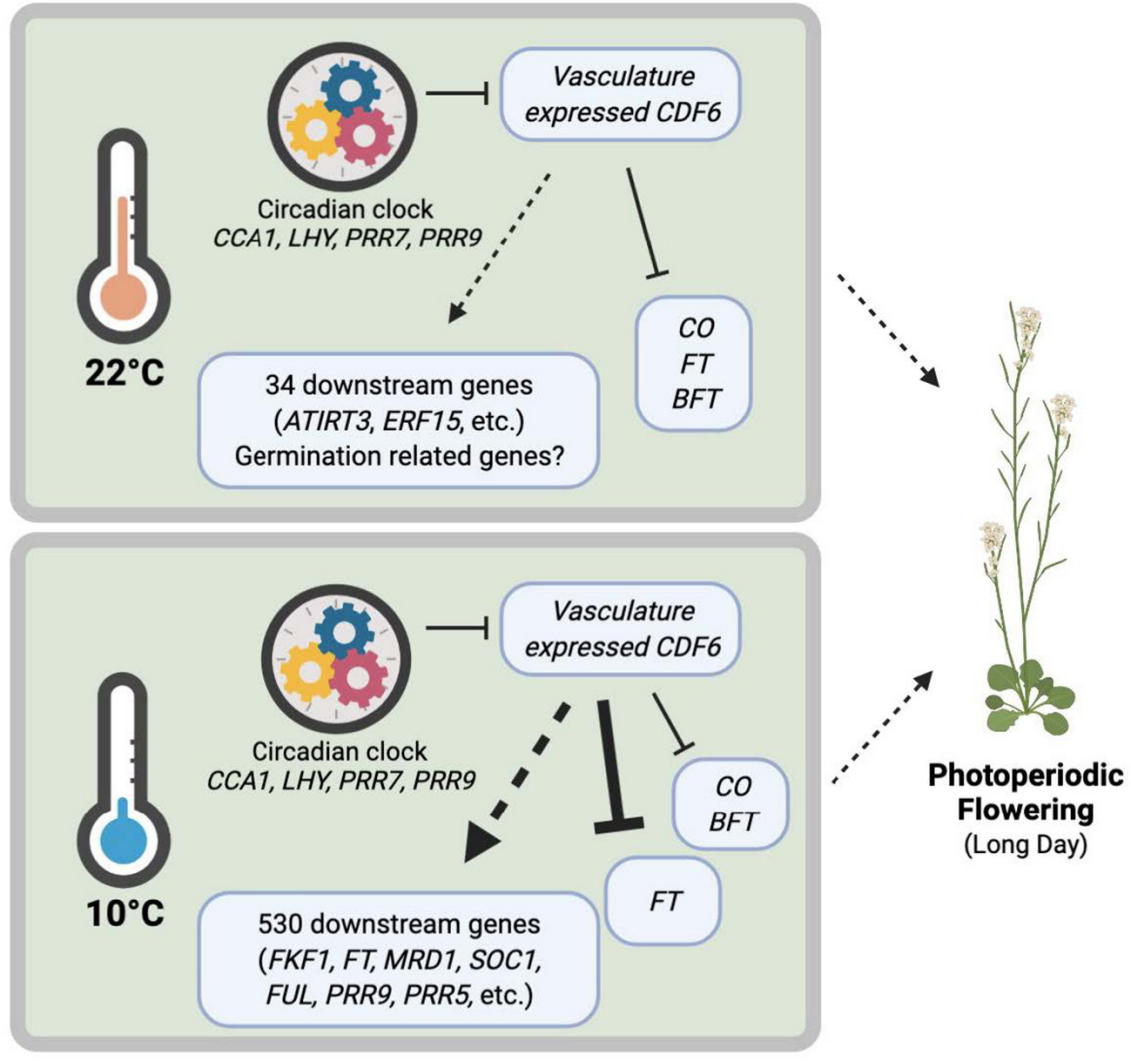
Clock-regulated, cold-induced, and vasculature-expressed *CDF6* finetunes plant growth and development under different temperatures. The circadian clock regulates *CDF6* transcript accumulation in the vasculature at specific times of the day. During ambient temperature, *CDF6* regulates *FT, CO, BFT*, and at least 34 other downstream genes (16 in *cdf6*-WT and 18 in *SUC2::CDF6*-WT), including *ATIRT3* and *ERF15*, which contribute to germination and flowering phenotypes. During cold temperatures, *CDF6* regulates 530 downstream genes and represses *FT, CO*, and *BFT* in the vasculature/phloem companion cells to control photoperiodic flowering. Model created with BioRender.com.

Here, we investigate the clock regulation of a poorly characterized member of the *CDF* family, *CDF6*, during ambient and cold temperatures. We find that CCA1 represses *CDF6* transcript accumulation during ambient temperature and gates the accumulation of *CDF6* during moderate cold stress (Figure 5). To better understand the clock regulation of *CDF6*, we utilized the FIMO tool to scan the 500 bp upstream of each DOF-TF family member for motifs associated with clock TF binding (Grant et al., 2011). This analysis identified that proximal promoter regions of ~39% of the DOF-TFs contain a full or partial evening element or CCA1-binding site, whereas none of the DOF-TF promoters contain a primary G-box motif, the element which is associated with PRR binding (Supplementary Dataset 5). This may explain the low number of DOF family members in PRR7 (~5%), PRR9 (~11%), and TOC1 (0) ChIP datasets compared to the CCA1 (~25%) and LHY (~14%) ChIP datasets, although it is important to note the CCA1 and LHY datasets also identified many more target genes than the PRR datasets (Huang et al., 2012; Liu et al., 2013, 2016; Nagel et al., 2015; Kamioka et al., 2016; Adams et al., 2018) (Supplementary Dataset 5). We also considered that clock genes may not be the only upstream regulators of *CDF6*, therefore, we utilized the DAP-sequencing database to identify TFs that may bind to the *CDF6* promoter region (O'Malley et al., 2016). This revealed 142 unique gene IDs/TFs that bind *in vitro* along the *CDF6* promoter, gene body, or UTRs (Supplementary Dataset 6). Of these, ~12% were DOF-TFs (including *CDF3, CDF4*, and *CDF5*), suggesting that these CDFs may regulate *CDF6* or other *DOF-TFs*. The finding that *CDF6* expression is significantly downregulated in *35S::CDF3* lines further supports the conclusion that CDF3 modulates *CDF6* transcription (Corrales et al., 2017). Finally, ~20% of the TFs identified in the DAP-sequencing analysis were also significantly mis-regulated in our generally cold-responsive dataset (FDR < 0.05), implying that some of these TFs may also play a role in the cold regulation of CDF6 (Supplementary Dataset 6). Future work to investigate whether these TFs play a role in modulating the cold response of *CDF6* and other *DOFs* is needed.

The results of this study indicate that *CDF6* participates in seed germination regulation during ambient temperature and photoperiodic flowering during cold stress (Figure 2). Other DOF-TFs function in vascular system development and germination; however, with the exception of CDF4, the *CDFs* have not been previously associated with germination (Le Hir and Bellini, 2013). Our transcriptomic analysis revealed that *IRT3, ERF15*, and 16 other DEGs may contribute to the observed germination phenotype (Figure 5; Supplementary Figure 4). Of note, our dataset was generated under a later developmental stage than when germination was phenotypically assessed, thus future work at the same developmental stage could elucidate any additional DEGs that may contribute to the delayed germination in *SUC2::CDF6*.

Our study supports a greater role for *CDF6* function under cold vs. ambient temperature to broadly control plant growth and development. We identified a number of flowering-associated genes that have altered expression in vasculature-expressed *CDF6* during cold stress, four (*FT, BFT, PRR9*, and *PRR5*) of which are also expressed in the PCC (Figure 3; Supplementary Figures 7A–D) (Mustroph et al., 2009). Of note, single-cell sequencing data in Arabidopsis shows that *CDF6* may also be expressed in phloem parenchyma, xylem, and even mesophyll cells (Mustroph et al., 2009; Kim et al., 2021). We also identified a number of GO terms associated with responses to stress, development, and metabolism (Figure 3). While the GO terms corresponding to the response to stress and development corroborate our earlier findings for *CDF6* during cold stress and in relation to both the observed germination and flowering phenotypes, the metabolism GO terms are interesting, as other CDFs have been previously implicated in metabolic regulation. A transcriptomic analysis of *35S::CDF3* indicates the enrichment of metabolism GO terms, and more specifically, a metabolic analysis indicates that *CDF3* over-expression impacts the metabolism of sugars and amino acids (Corrales et al., 2017). While the results of the GO analysis for both *SUC2::CDF6* and *35S::CDF3* yielded similar enriched terms, only a single gene is shared between the DEGs in the *CDF3* (531) and *CDF6* (18) datasets. The shared gene, *AT4G01390*, is significantly downregulated in *SUC2::CDF6* and upregulated in *35S::CDF3*. Based on public annotation, *AT4G01390* contains a MATH [meprin and TRAF (tumor necrosis factor receptor-associated factor) homology] domain which is found in proteins involved in several of the GO terms shared between the two datasets, such as stress responses, plant development, signaling, and metabolism (Oelmuller et al., 2005; Inzé et al., 2012; Qi et al., 2021). Of note, the lack of significant overlap between the two datasets is not surprising given the differences in tissue-specific expression (*35S::CDF3* vs. *SUC2::CDF6*), experimental design, and analysis. Together, this finding indicates that *CDF3* and *CDF6* may work similarly but independently to integrate temperature signals to alter metabolism and development.

Next, we observed that vasculature-expressed *CDF6* results in altered *FT, CO*, and *BFT* accumulation across the 24-h period at both 22 and 10°C, but notably during continuous cold on long days (Figure 4). Specifically, we found that *FT* shows higher accumulation during the cold from ZT20 to ZT36, while *CO* is higher at ZT24 and lower at ZT32 (Supplementary Figure 6). At first glance, this seems contrary to a previous work, which indicates that lower temperatures decrease *FT* accumulation and delay flowering (Song et al., 2013). However, the impact of temperature on *FT* accumulation is highly dependent on the timing of the temperature stress and the photoperiod length (Kinmonth-Schultz et al., 2016; Krahmer et al., 2019). For example, when plants are grown on ambient long days with cool nights, *CO* has higher transcript accumulation at dawn (ZT0) and no change at midday (ZT8) or dusk (ZT16), while *FT* is higher at dawn with a decrease or no change at midday and dusk when compared to the constant ambient temperature control conditions (Kinmonth-Schultz et al., 2016). In constant light, *FT* exhibits increased transcript abundance during cold treatment (Schwartz et al., 2009). *FT* is dynamically controlled during cold temperature exposure by multiple regulators, including *FLC, SHORT VEGETATIVE PHASE (SVP), HIGH EXPRESSION OF OSMOTICALLY RESPONSIVE GENES1 (HOS1), miR156, miR172*, and sequestration of FT, through its interaction with phospholipid phosphatidylglycerol (PG) at cellular membranes (Song et al., 2013; Susila et al., 2021). Our study shows for the first time that a *CDF* family member contributes to the differential transcript accumulation of key photoperiod regulators (*FT, CO*, and *BFT*) during cold stress. Additionally, ectopic expression of *CDF6* with *pSUC2* results in the differential expression of 34 and 530 downstream genes under ambient and cold temperatures, respectively (Figure 5). We conclude that CDF6 directly or indirectly regulates the transcription of numerous genes, particularly at low temperatures.

Taken together, our data suggest that vasculature-expressed *CDF6* plays a role in regulating photoperiodic flowering during cold stress, and some of this regulation involves functional redundancy with other *CDFs*. We provide new insights on the regulatory relationship between *CDF6* and the clock, cold stress, and plant development. As climate change continues to cause erratic weather events, the precise regulation of photoperiod flowering components in specific cell types during cold temperatures should be further explored as a tool to combat potential crop losses.

## Data Availability Statement

The datasets presented in this study can be found in online repositories. The names of the repository/repositories and accession number(s) can be found in the article/Supplementary Material.

## Author Contributions

DN conceived the project. EB and DN designed and performed experiments, analyzed the data, and wrote the manuscript. GG, ML, and TI generated and confirmed the plant materials. EB, TI, and DN revised and edited the manuscript. All authors contributed to the article and approved the submitted version.

## Funding

This work was supported by an NSF Early Career Award (IOS 1942949) to DN and in part by the National Institute of Health (R01GM079712) to TI.

## Conflict of Interest

The authors declare that the research was conducted in the absence of any commercial or financial relationships that could be construed as a potential conflict of interest.

## Publisher's Note

All claims expressed in this article are solely those of the authors and do not necessarily represent those of their affiliated organizations, or those of the publisher, the editors and the reviewers. Any product that may be evaluated in this article, or claim that may be made by its manufacturer, is not guaranteed or endorsed by the publisher.
