# Supplementary material for: Clock-Controlled and Cold-Induced CYCLING DOF FACTOR6 Alters Growth and Development in Arabidopsis
Source: Front Plant Sci. 2022 Jul 26;13:919676. doi: 10.3389/fpls.2022.919676 (PMC9361860; doi:10.3389/fpls.2022.919676)
Supplement: Supplementary file 1 [file Table_1.docx]

**Supplementary Table 1: PCR Conditions and Primer Sequences used in genotyping and plasmid construction PCR and qRT-PCR.**

For qRT-PCR conditions were 95°C for 3 min, 40 cycles at 95°C for 15 sec and 60 °C for 1 min, 95°C for 10 sec, and melt curve from 65°C to 95°C with 0.5°C increments. *Isopentenyl-diphosphate delta-isomerase II (IPP2)* was used as the normalization control.

*LHY-F:* 5’-CAACAGCAACAACAATGCAACTAC-3’

*LHY-R*: 5’-AGAGAGCCTGAAACGCTATACG-3’

*CCA1-F:* 5’-CAGCTCCAATATAACCGATCCAT-3’

*CCA1-R:* 5’-CAATTCGACCCTCGTCAGACA-3’

*CDF6qPCR-F:* 5’-GACTTGTATTGTCAGTAACAGATTGG-3’

*CDF6qPCR-R:* 5’-TGGCTGGACAATTACACCG-3’

*IPP2-F:* 5’-GTATGAGTTGCTTCTGGAGCAAAG-3’

*IPP2-R:* 5’-GAGGATGGCTGCAACAAGTGT-3’

*CO-F:* 5’-CTACAACGACAATGGTTCCATTAAC -3’

*CO-R:* 5’-CAGGGTCAGGTTGTTGC -3’

*FT-F:* 5’-CTGGAACAACCTTTGGCAAT -3’

*FT-R:* 5’-TACACTGTTTGCCTGCCAAG -3’

*BFT- F*: 5’-CGCCGGAAACTAGAGAGTGT-3’

*BFT- R*: 5’-GTTGGGCGTTGAAGTAAACA-3’

*CDF6pro-F:* 5’-CACCGTTCTGTTTCAGAAGCAAGAATTT -3’

*CDF6cds-R:* 5’-GGCAAGATCTATGAACTTCAGAGA -3’

*SALK_010734-F*: 5’ -TCGGATTTTGAAAGGTTGTTG - 3’

*SALK_010734-R*: 5’ -GTTACTTCCTCCCCAAGCATC - 3'

*SALK_010734qPCR-F:* 5’- GACATTACACTTCAGCATTTCCA -3’

*SALK_010734qPCR-R:* 5’- ATCTATCTTATTTATATACCACAATCCC -3’
