# Supplementary material for: Clock-Controlled and Cold-Induced CYCLING DOF FACTOR6 Alters Growth and Development in Arabidopsis
Source: Front Plant Sci. 2022 Jul 26;13:919676. doi: 10.3389/fpls.2022.919676 (PMC9361860; doi:10.3389/fpls.2022.919676)
Supplement: Supplementary file 2 [file Data_Sheet_1.docx]

**Supplementary Figures**

Clock controlled and cold-induced *CYCLING DOF FACTOR6* alters growth and development in Arabidopsis

**Authors**

Emily J. Blair^1^, Greg S. Goralogia,^2,3^, Matthew J. Lincoln^2,4^, Takato Imaizumi^2^, and Dawn H. Nagel^1*^


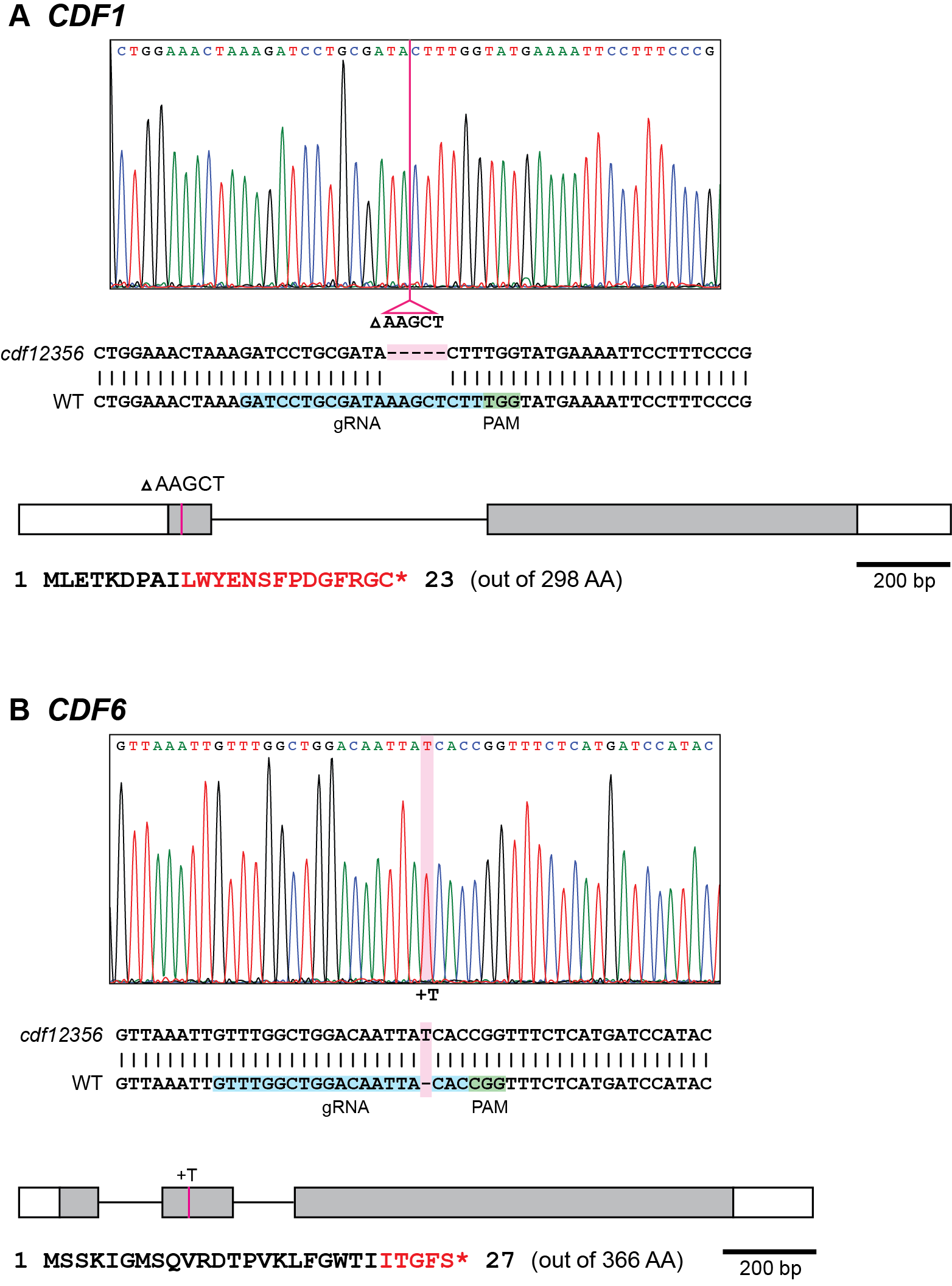


**Supplementary Figure 1: CRISPR/Cas9-induced mutations in *CDF1* and *CDF6* in the *cdf12356* mutant. (A)** A chromatogram of the region where mutations exist in the *CDF1* gene in the *cdf12356* mutant is presented. The nucleotide alignment of the same regions between the *cdf12356* and WT is shown under the chromatogram. The position of 5-base-pair deletion in *CDF1* in the *cdf12356* is highlighted in pink in the alignment. The positions of the gRNA and the PAM sequences are also highlighted. The position of the mutation is indicated in the diagram of the *CDF1* gene structure. The white and gray boxes represent UTRs and exons, respectively, and the bars connecting boxes are introns. The deduced amino acid sequences in the *cdf12356* are shown. The amino acid sequences that differed from the WT CDF1 sequences are highlighted in red. **(B)** The information regarding the mutation in *CDF6* in the *cdf12356* mutant. The chromatogram of the mutation in CDF6 in the *cdf12356* mutant, the nucleotide sequence alignment, the schematic diagram of the *CDF6* gene, and the deduced amino acid of resulting CDF6 protein are shown. The detailed information of the figures is the same as that of **(A)**.


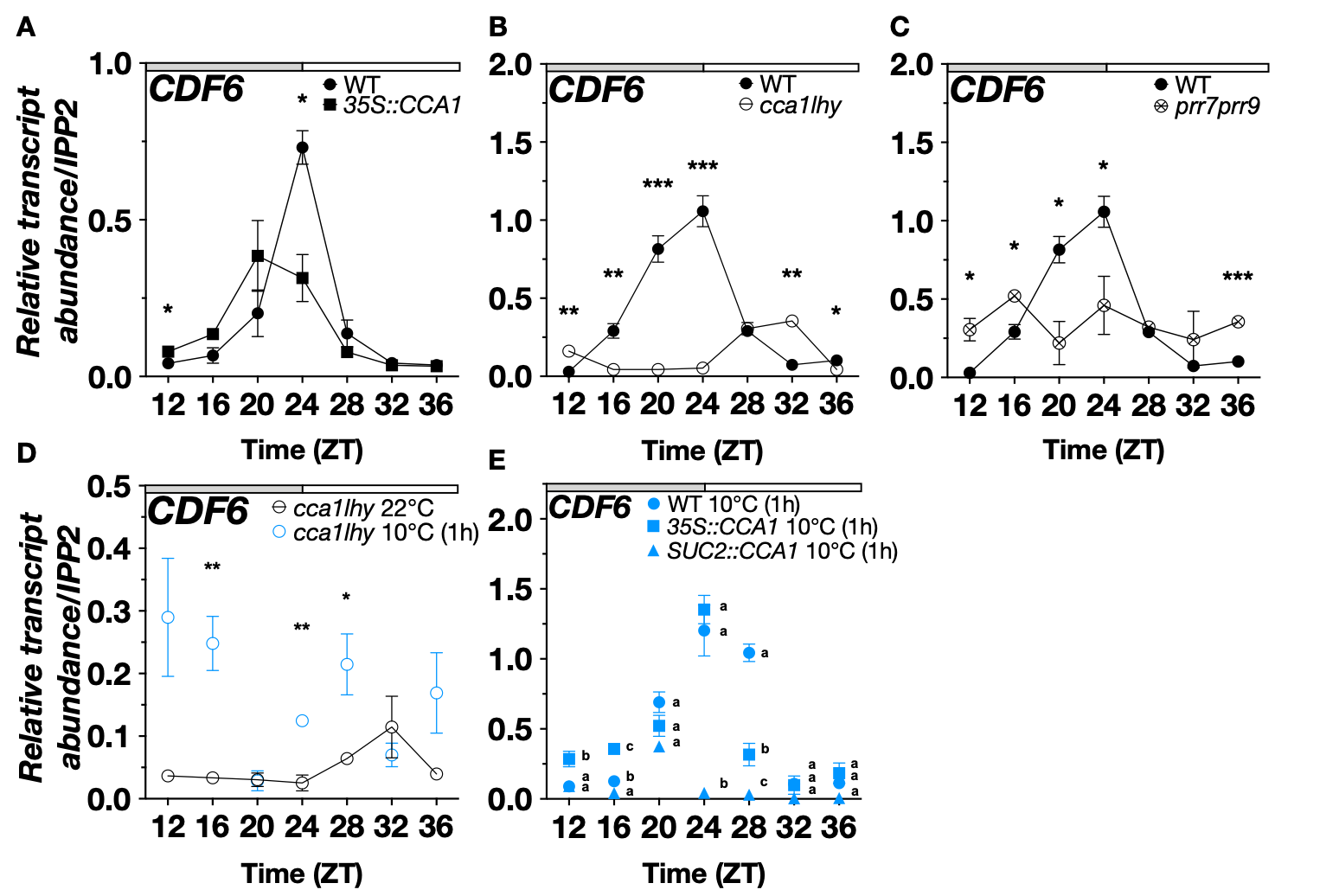


**Supplementary Figure 2: *CDF6* is regulated by morning and day-phased clock components.** qRT-PCR of *CDF6* in WT, **(A)** *35S::CCA1*, **(B)** *cca1lhy*, and **(C)** *prr7prr9* seedlings grown in constant light (LL) for 2 days after 8 days of entrainment in 12 h light:12 h dark (LD) cycles. qRT-PCR of *CDF6* transcript abundance in continuous 22°C and 1 h of 10°C exposure in (D) *cca1lhy* and (E) after 1 h of 10°C exposure in WT, *35S::CCA1* and *SUC2::CCA1*. Seedlings were grown as in (A-C). Plants were sampled every 4 h for 24 h. Gray and white bars indicate the subjective night and day periods, respectively. Time (ZT) represents hours. mRNA levels are normalized to *IPP2* (mean values ± SE, n=3, ***P ≤ 0.001; **P ≤ 0.01; *P ≤ 0.05, unpaired student t-test). (E) Lowercase letters indicate statistical significance determined by 1-way ANOVAs performed at each time point (mean values ± SE, n=3, 1-way ANOVA with Brown-Forsythe multiple comparison test).


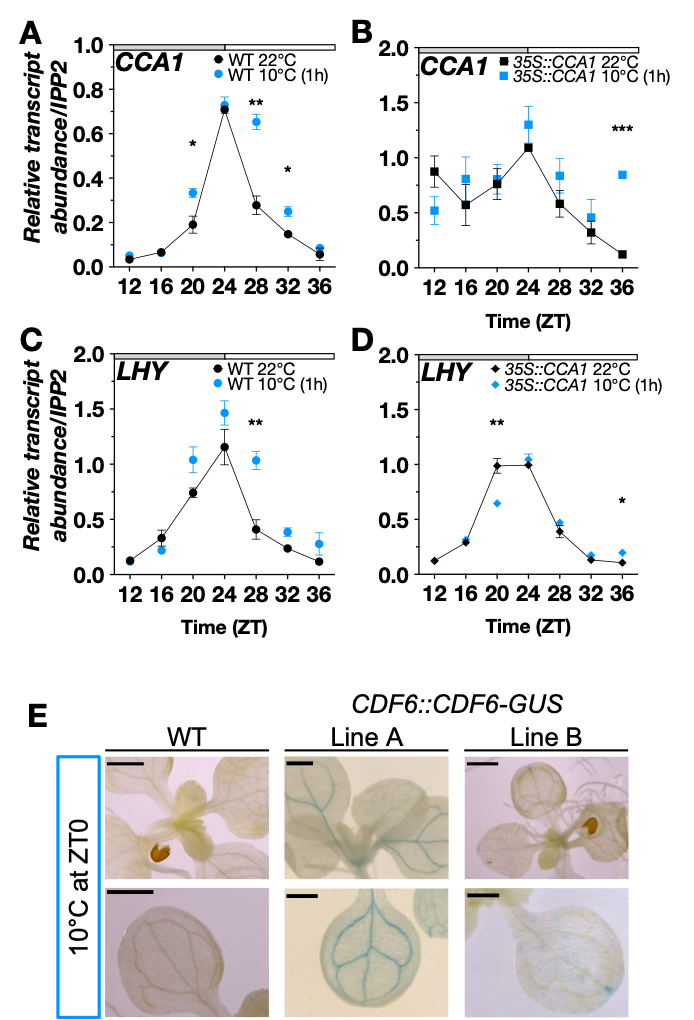


**Supplementary Figure 3: *CCA1* and *LHY* have time of day specific response to cold stress and CDF6 protein localizes to vasculature during cold stress.** qRT-PCR of **(A and B)** *CCA1* and **(C and D)** *LHY* transcript levels in WT (Col-0) and *35S::CCA1* seedlings grown in constant light (LL) for 2 days after 8 days entrainment in 12 h light:12 h dark (LD) cycles. Plants were sampled every 4 h for 24 h at constant 22°C or after a 1 h 10°C exposure. Gray and white bars indicate the subjective night and day periods, respectively. mRNA levels are normalized to *IPP2* (mean values ± SE, n=3, ***P ≤ 0.001; **P ≤ 0.01; *P ≤ 0.05, unpaired student t-test). **(E)** GUS activity indicates expression pattern of CDF6 at ZT0 in 11 day old seedlings grown in long-days (16h light:8 h dark) and then exposed to 1 h 10°C treatment. Bars correspond to 500 µm.


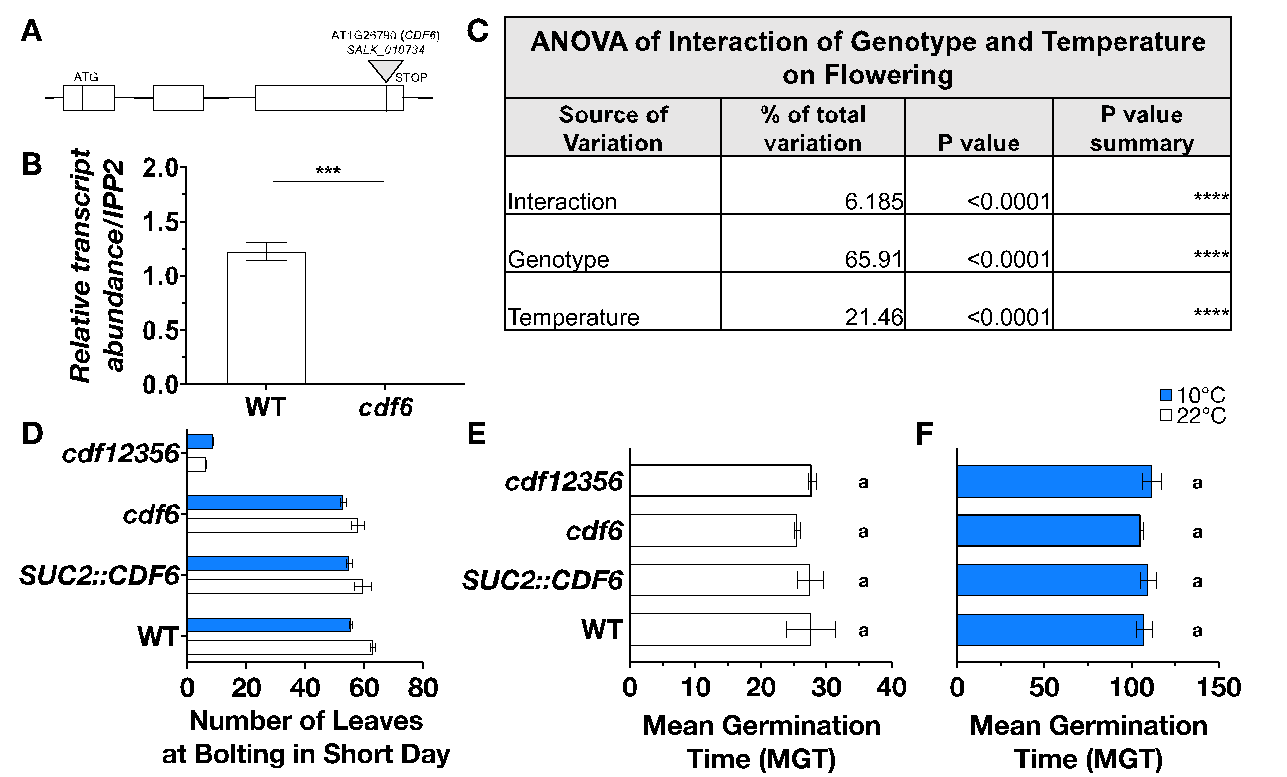


**Supplementary Figure 4: *CDF6* transcript abundance is downregulated in *cdf6* and dormancy does not change in *CDF6* mis-expression lines. (A)** Gene model with SALK T-DNA insertion mutant line (SALK_010734) for *CDF6* based on Araport11. **(B)** qRT-PCR of *CDF6* transcript accumulation in 10 day old seedlings grown in 12 h light:12 h dark (LD) cycles and sampled at ZT0. mRNA levels are normalized to *IPP2* (mean values ± SE, n=3, ***P ≤ 0.001; **P ≤ 0.01; *P ≤ 0.05, unpaired student t-test). **(C)** Results of the ordinary 2-way ANOVA performed on Figure 2A data. (**D**) Number of leaves at bolting for WT, *SUC2::CDF6*, *cdf6* and *cdf12356* plants grown in constant 22°C or 10°C in short day (8h light: 16h dark) conditions (mean values ± SE, n=1). Mean germination time for WT, *SUC2::CDF6, cdf6* and *cdf12356* plants grown in constant light (LL) after 2-3 nights of stratification **(E)** 22°C and **(F)** 10°C (mean values ± SE, 1-way ordinary ANOVA with Dunnett’s multiple comparison test).


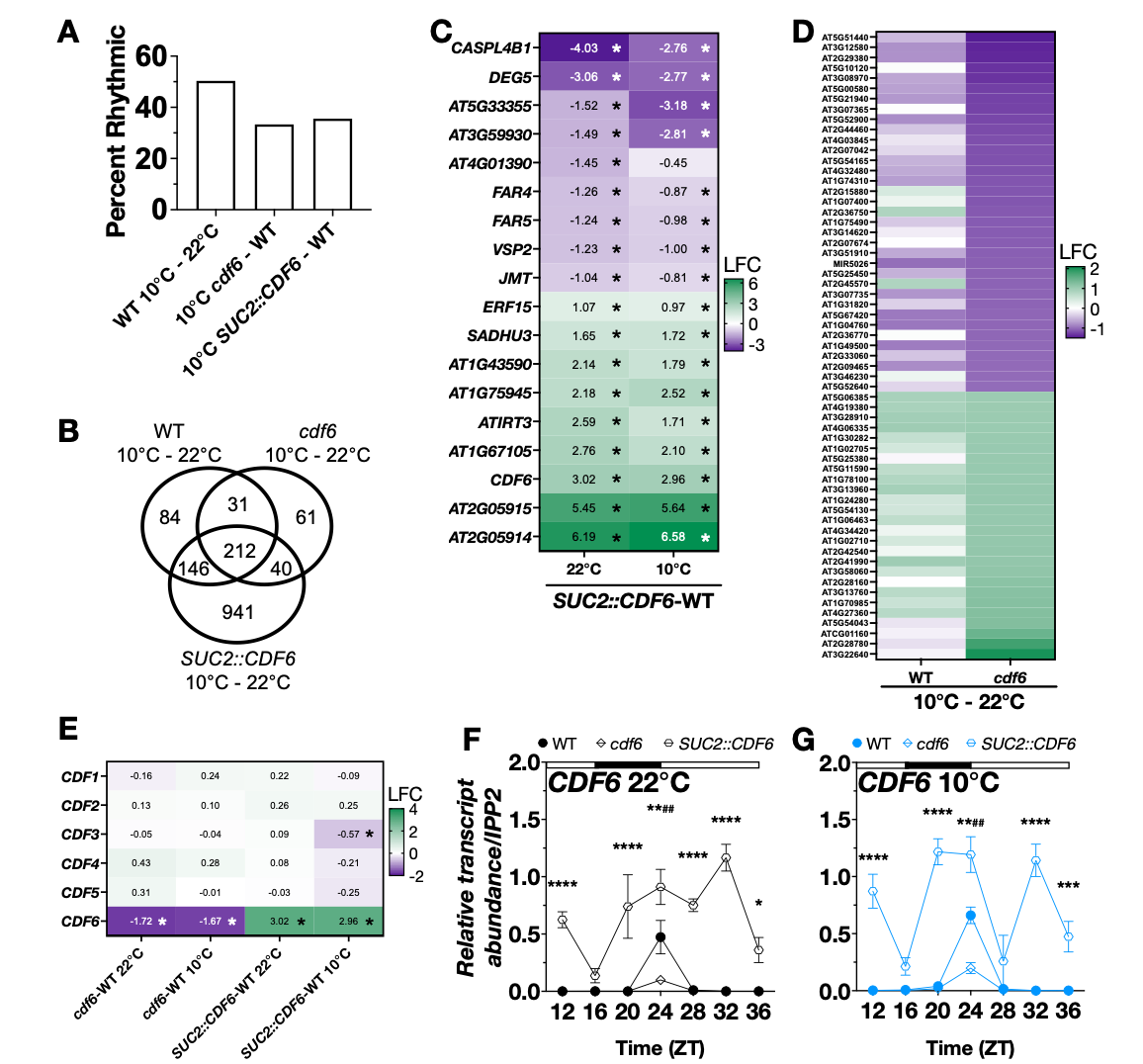


**Supplementary Figure 5: Genome-wide expression analysis reveals both cold-dependent and *CDF6-*dependent DEGs.** **(A)** Percentage of DEGs (defined as -1 > Log_2_ Fold Change (LFC) > 1 and False Discovery Rate (FDR) < 0.05) that exhibit rhythmic pattern of expression determined using the Phaser webtool (Mockler et al., 2007). **(B)** Number of shared and unique DEGs at 10°C versus 22°C in WT, *SUC2::CDF6,* and *cdf6*. (**C**) LFC for 18 genes that are differentially expressed in *SUC2::CDF6* compared to the WT at 22°C and 10°C. **(D)** LFC for the 61 DEGs in *cdf6* at 10°C compared to 22°C. (E) LFC for *CDF1, 2, 3, 4, 5,* and *6* in *cdf6* or *SUC2::CDF6* compared to WT at both 10°C and 22°C (*FDR <  0.05). qRT-PCR of *CDF6* transcript accumulation in 11 day old seedlings grown in long-day (16 h light: 8 h dark) at 22°C for 8 days followed by 2 days maintained at continuous 22°C **(F)** or transferred to continuous 10°C **(G)**. Plants were sampled every 4 h for 24 h. White and black bars indicate the day and night periods, respectively. mRNA levels are normalized to *IPP2* (mean values ± SE, n=3, ****P ≤ 0.0001; ***P ≤ 0.001; **/##P ≤ 0.01; *P ≤ 0.05; where * indicates a significant difference between WT and *SUC2::CDF6* while # indicates a significant difference between WT and *cdf6.*


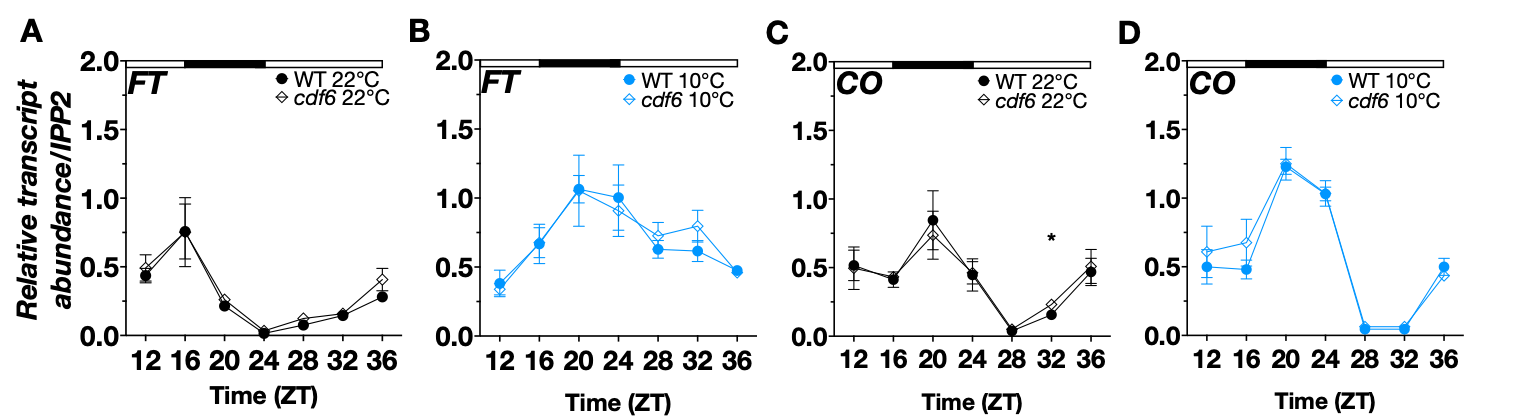


**Supplementary Figure 6: FT and CO response to cold in WT and *cdf6*.** qRT-PCR of **(A, B)** *FT* and **(C, D)** *CO* relative transcript abundance in WT and *cdf6* seedlings grown in long-day (16 h light: 8 h dark) conditions at 22˚C for 8 days followed by 2 days at continuous 22˚C (A,C) or 10˚C (B,D). Seedlings were sampled every 4 h starting at 12 h after dawn. White and black bars indicate the day and night periods, respectively. mRNA levels are normalized to *IPP2* (mean values ± SE, n=3; ***P ≤ 0.005, **P ≤ 0.01, *P ≤ 0.05; unpaired student t-test).


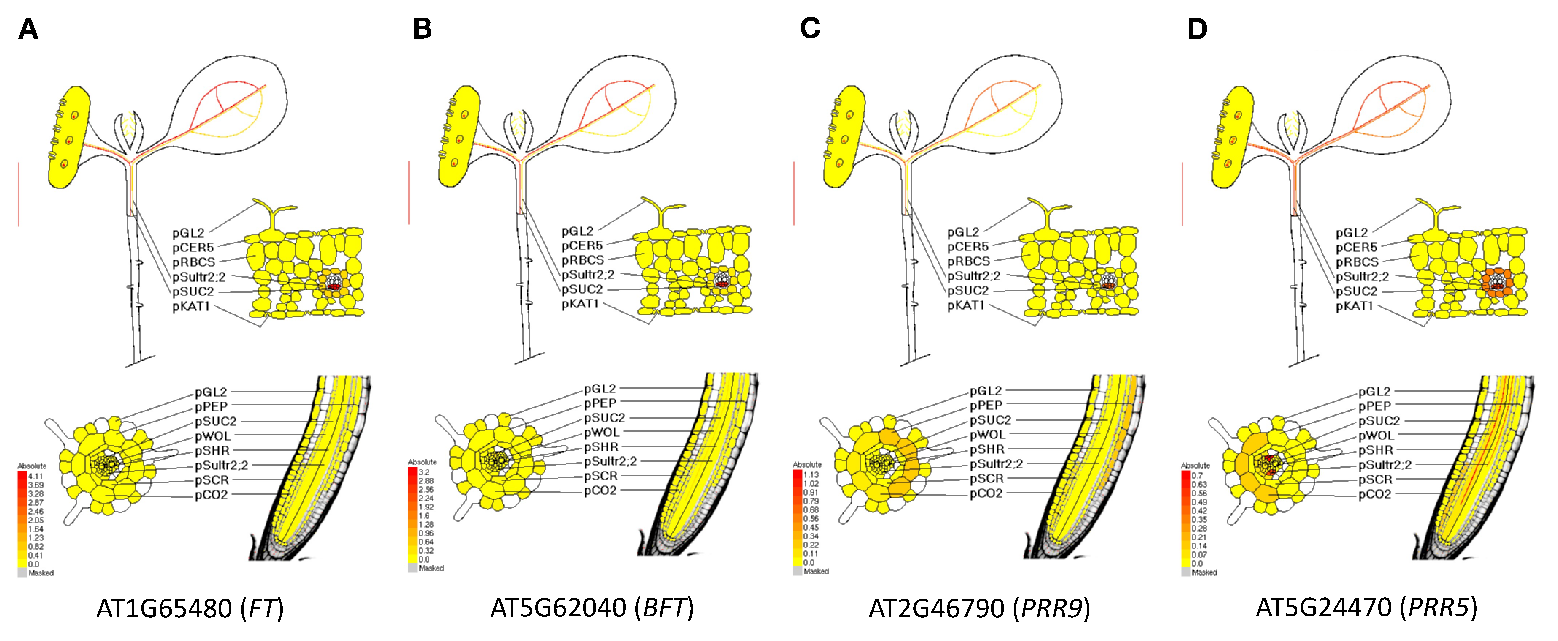


**Supplementary Figure 7:** **(A) *FT*, (B) *BFT*, (C) *PRR9*, and (D) *PRR5* transcripts primarily localize to the vasculature/phloem companion cells (pSUC2) in control conditions.** Pictograms were taken from http://efp.ucr.edu/ (Mustroph et al., 2009). Data represent signal values from microarray; scale bars are different between panels.
